# A novel SaeS-targeting antivirulence antagonist screened against methicillin-resistant *Staphylococcus aureus*

**DOI:** 10.3389/fcimb.2026.1867130

**Published:** 2026-07-08

**Authors:** Jiahao Yao, Duiyuan Ai, Huanhuan Duan

**Affiliations:** College of Food Science and Engineering, Gansu Agricultural University, Lanzhou, Gansu, China

**Keywords:** antivirulence, computational modeling, methicillin-resistant *Staphylococcus aureus*, quorum-sensing quenching, SaeRS two-component system

## Abstract

**Background:**

The SaeRS two-component system is a major virulence regulator in *Staphylococcus aureus*, and its sensor kinase SaeS represents a potential target for small-molecule intervention.

**Methods:**

In this study, we constructed a library of 1,093 compounds from PubChem and carried out structure-guided virtual screening against the SaeS kinase domain. This approach identified an amide-based candidate, designated Saeamid-1. Molecular docking and molecular dynamics simulations supported stable binding of Saeamid-1 within the nucleotide-binding region of SaeS, with a docking score of -9.884 kcal/mol.

**Results:**

The compound had only limited effects on bacterial growth. In contrast, it reduced hemolytic activity and biofilm biomass in USA300 by 65.74% and 88.77%, respectively. RT-qPCR analysis showed that Saeamid-1 reduced the expression of *saeR, saeS*, and the *SaeRS-*associated virulence gene *spa*.

**Conclusions:**

Taken together, these findings identify Saeamid-1 as a promising antivirulence candidate that targets SaeS in methicillin-resistant *Staphylococcus aureus* and provide a basis for further development of therapeutic strategies directed against the SaeRS pathway.

## 1 Introduction

**Figure 1:**
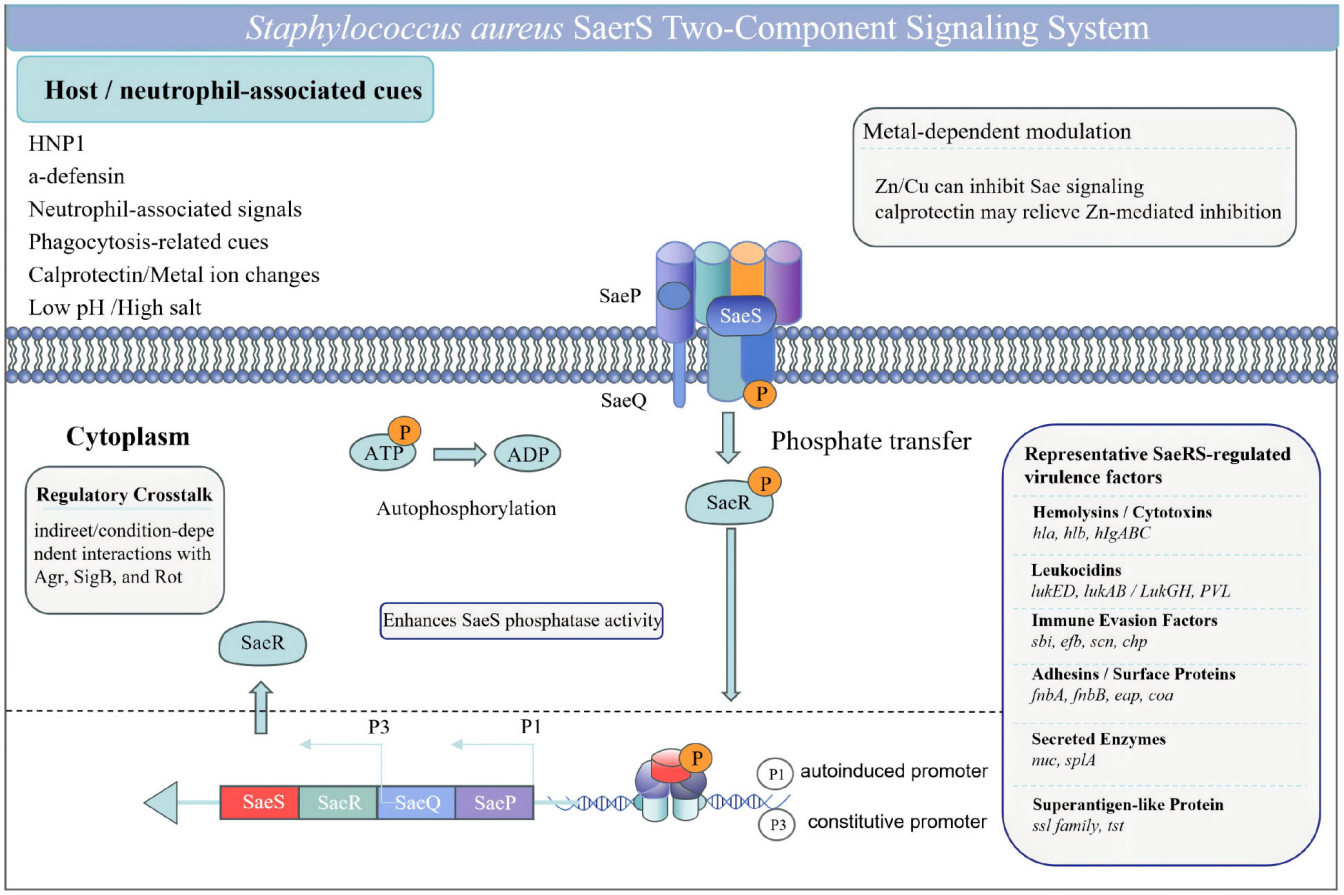
Schematic model of the SaeRS two-component signaling system in *Staphylococcus aureus*.

Methicillin-resistant *Staphylococcus aureus* (MRSA) is a major public health concern. MRSA can cause refractory and invasive infections and can also form biofilms on tissue surfaces and medical devices. These characteristics increase its tolerance to antibiotic treatment and enhance its ability to evade immune clearance (Xu et al., 2025). Therefore, virulence-targeting strategies have attracted considerable attention. The goal of these strategies is to modulate virulence rather than directly affect bacterial survival. The SaeRS two-component system is a major virulence regulatory system in *Staphylococcus aureus* (*S. aureus*) (Sun et al., 2010) (**Figure 1**). In this system, SaeS is the sensor histidine kinase, and SaeR is the response regulator. SaeS first undergoes autophosphorylation in response to a stimulating signal and then transfers the phosphate group to SaeR. Phosphorylated SaeR binds to SaeR-binding sites and activates transcription of target genes. SaeP and SaeQ can form a complex with SaeS, thereby enhancing its phosphatase activity (Jeong et al., 2012). SaeRS regulates multiple virulence genes, including hemolysins, leukocidins, and several surface-associated factors. Beyond classical toxin regulation, SaeRS is also functionally linked to biofilm-associated surface programs and cell wall turnover (Liu et al., 2015). Studies have shown that during biofilm development, SaeRS is linked to autolysis and remodeling processes involving fibronectin-binding protein A (FnBPA; fnbA) and the major autolysin AtlA. In addition, SaeRS acts together with the SarA-associated protease regulatory network to limit extracellular protease production, including aureolysin (Aur) and the SspA/SspB protease system. This effect can reduce protease-mediated degradation of key surface determinants, such as staphylococcal protein A (Spa) and fibronectin-binding proteins (FnBPs), thereby contributing to biofilm stability (Mrak et al., 2012). SaeRS also functions within a broader regulatory network that includes Agr, SigB, and Rot. Agr and SaeRS regulate overlapping but non-identical virulence outputs. Rot can cooperate with SaeRS for selected targets (Benson et al., 2012), whereas SigB influences Sae-related virulence regulation in a strain-dependent and condition-dependent manner (Goerke et al., 2005).

Although previous studies have supported the SaeRS pathway as an antivirulence target, small-molecule research on this system remains limited (Liang et al., 2006; Nygaard et al., 2018; Ekness et al., 2025). SKKUCS has been reported to repress the expression of Sae-associated virulence genes, reduce hemolytic activity, and interfere with the autokinase function of SaeS (Arya et al., 2023). More recent studies have further shown that small molecules targeting SaeR, such as HR3744 and its optimized analogue SAV13, can bind to the DNA-binding domain of SaeR and block the interaction between SaeR and DNA (Gao et al., 2023). Together, these findings indicate that the SaeRS pathway is druggable (Bem et al., 2015; Chen et al., 2022). However, systematic small-molecule development based on its structural features is still insufficient. In particular, inhibitors directed against SaeS remain limited in both number and chemotype diversity, and the structure-activity relationships and molecular mechanisms associated with the kinase pocket have not yet been fully defined (Ekness et al., 2025). To address this gap, we established a structure-based virtual screening workflow using SaeS structural information retrieved from the Protein Data Bank (PDB, https://www.rcsb.org/), combined with docking results generated by AutoDock Vina (version 1.2.7) and molecular dynamics simulations (MDS) to evaluate the stability, conformational behavior, and interaction persistence of candidate compounds within the binding pocket. Compounds prioritized through this workflow were then evaluated experimentally in the MRSA reference strain USA300 to determine their effects on bacterial growth, hemolytic activity, and biofilm formation. In addition, qPCR was performed to assess changes in the expression of key genes, including *saeR*, *saeS*, *spa*, *agrA*, and *agrC*. Through this integrated strategy, which combined computational screening with experimental validation, we aimed to identify a small molecule targeting SaeS with antivirulence activity and to provide mechanistic evidence linking its phenotypic effects to modulation of the SaeRS regulatory pathway.

## 2 Methods

### 2.1 Bacterial strain and culture conditions

Methicillin-resistant *Staphylococcus aureus* USA300 was used throughout this study and was obtained from Zhili Zhongte Biotechnology Co., Ltd. (Wuhan, China). The strain was stored as glycerol stocks at -80 °C. Before each experiment, the bacteria were revived, purified by passaging, and cultured overnight in tryptic soy broth at 37 °C with shaking. After two subcultures, the bacterial cultures were used for subsequent phenotypic and molecular assays.

### 2.2 Compound preparation and vehicle control

**Figure 2:**
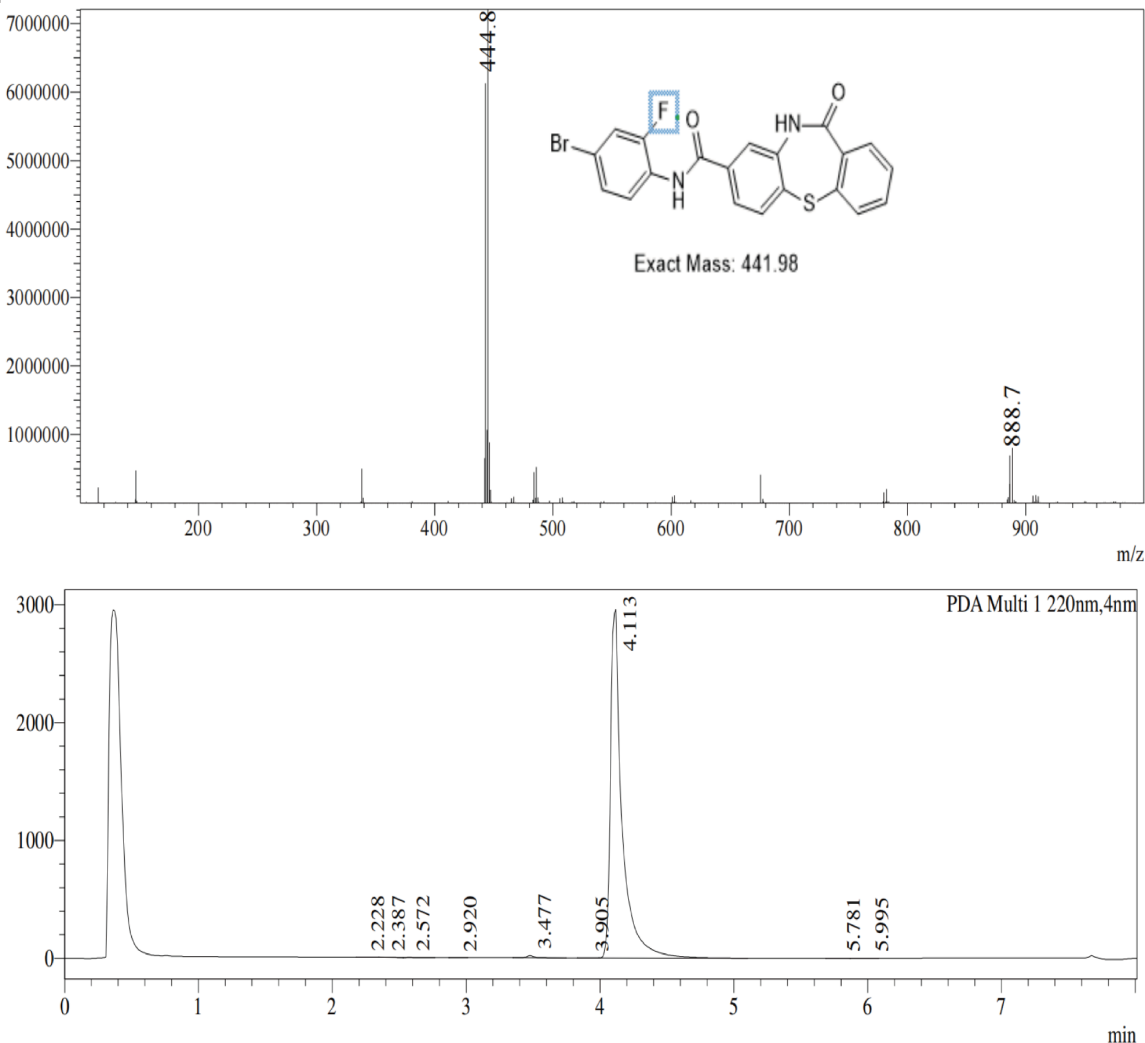
Mass spectrometric identification and HPLC analysis of Saeamid-1. A major ion peak at m/z 444.8 was detected in positive ESI mode, together with a weaker signal at m/z 888.7. HPLC analysis showed a dominant peak at 4.113 min, indicating high purity.

The test compound, an amide derivative with the chemical name N(4-bromo-2-fluorophenyl)-11-oxo-10,11-dihydrodibenzo[b,f][1,4]thiazepine-8-carboxamide, was obtained from WuXi AppTec (Shanghai, China) and dissolved in dimethyl sulfoxide to prepare a stock solution (**Figure 2**). The stock solution was sterilized by filtration through a 0.22 μm membrane and stored at -20 °C. For each experiment, the stock solution was diluted into the culture system to generate the required concentration gradient. The final concentration of dimethyl sulfoxide was maintained at no more than 1% v/v in all treatment groups. The vehicle control received an equal volume of dimethyl sulfoxide. Unless otherwise specified, each condition included three technical replicates, and the data are presented as the mean ± standard deviation.

### 2.3 Candidate library construction and virtual screening

The reported SaeS lead compound SKKUCS was used as the query structure for a PubChem two-dimensional similarity search (Kim S. et al., 2025). The Similar Compounds function was applied with a Tanimoto cutoff of 0.95 or higher. After removal of duplicate entries and structurally abnormal compounds, a curated candidate library containing 1,093 compounds was obtained. The SaeS kinase domain was then used as the receptor, and the nucleotide-binding region was defined as the docking site. Batch docking was performed using AutoDock Vina (version 1.2.7) (Eberhardt et al., 2021). Compounds were ranked according to their predicted binding affinities in kcal/mol, with more negative values indicating stronger predicted binding.

### 2.4 Molecular docking

The SaeS structure was retrieved from the AlphaFold Protein Structure Database (AlphaFoldDB entry: AF-Q2FIT5-F1; UniProtKB accession: Q2FIT5). This structure was used as a computed structural model. The kinase domain was extracted for docking analysis (Kim et al., 2025). Receptor preparation was performed using AutoDockTools (version 1.5.7), including structural inspection, addition of polar hydrogens, assignment of Gasteiger charges, and conversion of the receptor to PDBQT format. Ligand structures were retrieved from PubChem, energy-minimized, and prepared for docking by assigning Gasteiger charges and converting them to PDBQT format. The docking search space was defined around the nucleotide-binding region of SaeS. The grid box center was set to x = −12.603, y = 12.388, and z = −9.080, with dimensions of 33.75 Å × 15.00 Å × 15.00 Å along the x, y, and z axes, respectively. AutoDock Vina (version 1.2.7) was used to generate multiple binding poses (Eberhardt et al., 2021). The poses were initially screened on the basis of predicted binding affinity and conformational clustering. Selected poses were then inspected in PyMOL (version 3.1.4.1), and representative candidates were advanced to molecular dynamics simulations. The two-dimensional interaction diagram of the docking complex was generated using BIOVIA Discovery Studio Visualizer (Dassault Systèmes BIOVIA, 2021).

### 2.5 Molecular dynamics simulations

The docked complex of SaeS and the ligand was used as the starting structure for molecular dynamics simulations in GROMACS (version 2023.2) (Abraham et al., 2015). The protein was described using the CHARMM36m force field (Huang et al., 2017), and the ligand topology and parameters were generated separately using Sobtop [version 1.0 (dev3.1)] (Vanommeslaeghe and MacKerell, 2012). The system was solvated in a TIP3P water box with a minimum solute-to-box distance of 1.0 nm. Sodium chloride was then added to a final concentration of 0.15 M, and the system was neutralized. After energy minimization, equilibration was carried out under NVT conditions at 300 K and NPT conditions at 1 bar, with restraints applied to the protein backbone. A production simulation was then performed, for example, for 200 ns with a 2 fs time step. The trajectories were analyzed for root-mean-square deviation, root-mean-square fluctuation, radius of gyration, hydrogen-bond occupancy, salt-bridge occupancy, hydrophobic contacts, and ligand stability within the binding region.

### 2.6 Growth assay

Overnight cultures were inoculated into fresh tryptic soy broth at 1% v/v and cultured at 37 °C with shaking. The compound was added when the culture reached an OD600 of 0.3, and the final concentration of dimethyl sulfoxide was maintained at no more than 1% v/v. A vehicle control containing the same volume of dimethyl sulfoxide was included. When required, an antibiotic-treated positive control was also included. Cultures were dispensed into 96-well plates and incubated at 37 °C. Optical density at 600 nm was recorded every 30 min for 24 h.

### 2.7 Hemolysis assay

Cultures were grown to an OD600 of 0.3 before compound treatment. The final concentration of dimethyl sulfoxide was maintained at no more than 1% v/v. The cultures were then incubated with shaking until the OD600 reached 0.8. Cell-free supernatants were collected by centrifugation. For each reaction, 100 μL of supernatant was mixed with 150 μL of phosphate-buffered saline and 250 μL of a 4% suspension of defibrinated rabbit erythrocytes. The mixtures were incubated at 37 °C for 1 h and then centrifuged. Hemoglobin release was quantified by measuring the absorbance of the supernatant at 50 nm (Ridder et al., 2021). The untreated group was defined as 100% hemolysis, and phosphate-buffered saline plus erythrocytes served as the blank control.

### 2.8 Biofilm quantification

Overnight bacterial cultures were diluted to an initial OD600 of 0.01. Aliquots of 1 mL were dispensed into 24-well plates. Glucose was added to a final concentration of 0.25% when required. Compounds were then added to generate the desired concentration gradient. The plates were incubated statically at 37 °C for 16 h. After incubation, the supernatant was removed, and the wells were gently washed with phosphate-buffered saline. The biofilms were then fixed, stained with crystal violet, and washed to remove unbound dye. The bound dye was dissolved in 30% acetic acid by volume. Absorbance was measured at 595 nm. Relative biofilm biomass was calculated after subtraction of the blank value (Borowicz et al., 2023).

### 2.9 RT-qPCR

Cultures were grown to an OD600 of 0.3 before compound treatment, and the final concentration of dimethyl sulfoxide was maintained at no more than 1% v/v. The cultures were then incubated with shaking until the OD600 reached 0.8. Cells were immediately harvested for total RNA extraction. The RNA was reverse-transcribed into complementary DNA. Quantitative PCR was carried out using SYBR Green chemistry, with gyrB as the reference gene. Relative transcript levels of some key SaeSR correlational genes were calculated using the 2^^−ΔΔCt^ method, with the untreated group serving as the calibrator (**Table 1**) (Livak and Schmittgen, 2001).

**Table 1:** Primers for SaeSR-associated genes.

Primers	Sequences (5’-3’)
*AgrA*-F	ACGTGGCAGTAATTCAGTGT
*AgrA*-R	ATGGGCAATGAGTCTGTGAG
*AgrC*-F	GCTGATGATATACCACGAATTC
*AgrC*-R	GACCTAAACCACGACCTTCA
*SaeS*-F	CTAATCCAGAACCACCGTTT
*SaeS*-R	GCGATGAAGGTATTGGCATTATAC
*SaeR*-F	CCGCTAGTTGTCGTTGTTACT
*SaeR*-R	CCCACTTACTGATCGTGGATG
*Hla-F*	CTGTCGCTAATGCCGCAGATTCTG
*Hla-R*	CTTCTTCGCTATAAACTCTATATTGACCAGC
*FnbA*-F	TACCCGTTTCCACTTTCGC
*FnbA*-R	GGCTACACAAAATCAAGTCGC
*Spa*-F	ATGTCGTTAAACCTGGTGAT
*Spa*-R	CTTTGTTAGCATCTGCATGG
*gyrB*-F	AAGTGCGTCAAGTTGTAGAT
*gyrB*-R	TCTAGAGTCACGACCAGATT

### 2.10 Data analysis

Data are presented as the mean ± standard deviation (SD) from at least three independent experiments. Statistical analyses were performed using IBM SPSS Statistics 27. Differences among multiple groups were evaluated by one-way analysis of variance (ANOVA). When a significant overall difference was detected, pairwise multiple comparisons were performed to compare differences between groups. Differences were considered statistically significant at P < 0.05.

## 3 Results

### 3.1 Virtual screening and candidate prioritization

**Figure 3:**
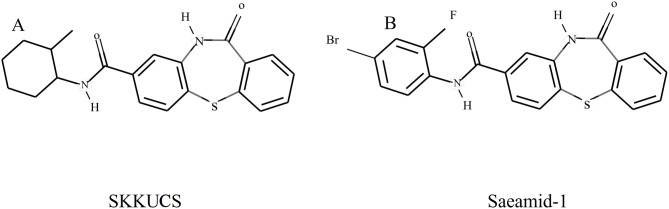
2D chemical structures of the studied compounds: **(A)** SKKUCS and **(B)** Saeamid-1.

We used the reported SaeS lead scaffold SKKUCS as the starting point for hit expansion. A two-dimensional similarity search was conducted in PubChem, and after duplicate removal and structural quality filtering, a curated screening library of 1,093 compounds was obtained (Carlsson and Luttens, 2024). The SaeS kinase domain was then used as the receptor for batch docking, and all candidates were ranked by predicted binding affinity within a structure-guided workflow (Ma et al., 2024). This workflow generated the prioritized hit list shown in **Table 2**, from which Saeamid-1 was selected for subsequent docking analysis, molecular dynamics evaluation, and *in vitro* validation (**Figure 3**). Saeamid-1 is a halogen-substituted aryl carboxamide built on a dibenzo[b,f][1,4]thiazepinone core, with a molecular formula of C20H12BrFN2O2S and a molecular weight of 443.30 g/mol. The compound contains a rigid fused heterocyclic scaffold linked to an aromatic amide moiety. Its bromine and fluorine substituents enhance hydrophobicity, whereas the amide and carbonyl groups provide limited polarity for local interactions. Its hydrogen-bond donor count is 2, the hydrogen-bond acceptor count is 4, it has no formal charge, and its topological polar surface area is 83.5 Å². These structural features support its accommodation within the hydrophobic nucleotide-binding pocket of SaeS and provide a reasonable basis for its selection for further docking, molecular dynamics, and *in vitro* studies.

**Table 2:** Top-ranked candidate compounds from virtual screening.

Compound	binding energy	PubChem CID
N-(4-bromo-2-fluorophenyl)-11-oxo-10,11-dihydrodibenzo[b,f][1,4]thiazepine-8-carboxamide	-9.884	46260653
6-keto-N-[3-(2-ketopyrrolidino)propyl]-5H-benzo[b][1,4]benzothiazepine-3-carboxamide	-9.665	9551335
N-(furan-2-ylmethyl)-6-oxo-5H-benzo[b][1,4]benzothiazepine-3-carboxamide	-9.661	4577045
6-keto-N-[3-(2-ketopyrrolidino)propyl]-5H-benzo[b][1,4]benzothiazepine-3-carboxamide	-9.579	9551335
N-cyclopentyl-6-oxo-5H-benzo[b][1,4]benzothiazepine-3-carboxamide	-9.439	4097179
N-cyclopropyl-6-oxo-5H-benzo[b][1,4]benzothiazepine-3-carboxamide	-8.958	11957178
1-benzyl-N-cyclohexyl-3,6-dihydro-2H-pyridine-4-carboxamide	-8.85	53051108
(6aS)-N-cyclohexyl-6-oxo-5,6a,7,8,9,10-hexahydropyrido[1,2-a]quinoxaline-3-carboxamide	-8.658	92726272

### 3.2 Predicted structure of SaeS

**Figure 4:**
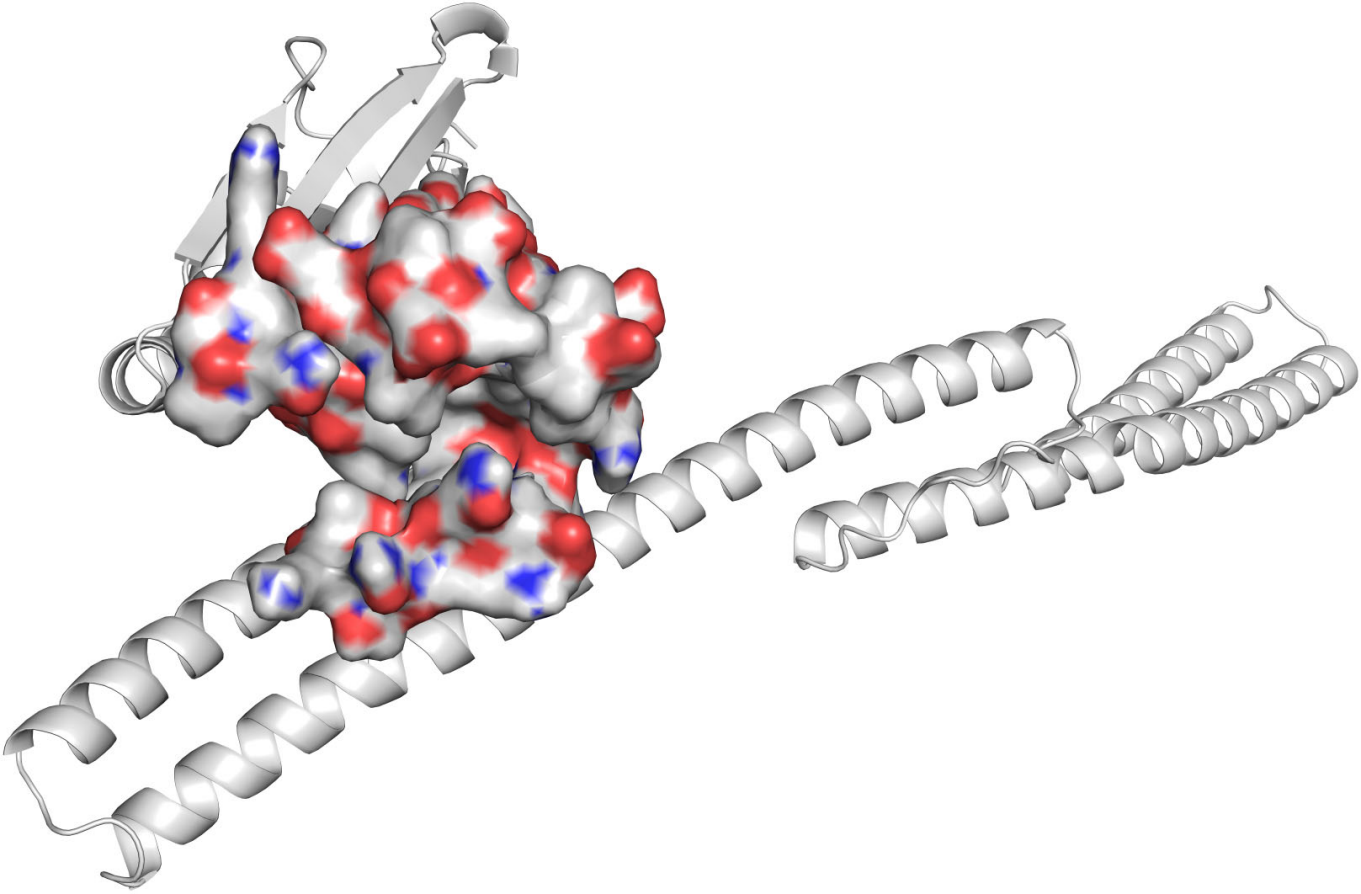
Overall predicted structure of SaeS with the binding pocket highlighted.

SaeS (Q2FIT5 · SAES_STAA3) is a 351 amino acid membrane-associated histidine kinase that functions as a key sensor protein in the SaeRS two-component system of *S. aureus*. This kinase catalyzes the following reaction: ATP + protein L-histidine = ADP + protein N-phospho-L-histidine. It phosphorylates the conserved histidine residue and belongs to enzyme class EC 2.7.13.3. It contains two transmembrane helices (position 9–29 and 40-60 in the polypeptide chain), a short extracellular loop, a HAMP-like region (positions 61-114 in the polypeptide chain), and a C-terminal kinase domain (positions 129-348 in the polypeptide chain, **Figure 4**). Previous studies have suggested that the putative nucleotide-binding pocket is located in the C-terminal kinase domain, indicating that this domain is likely important for signal transduction and phosphotransfer-related activity.

### 3.3 Molecular docking

**Figure 5:**
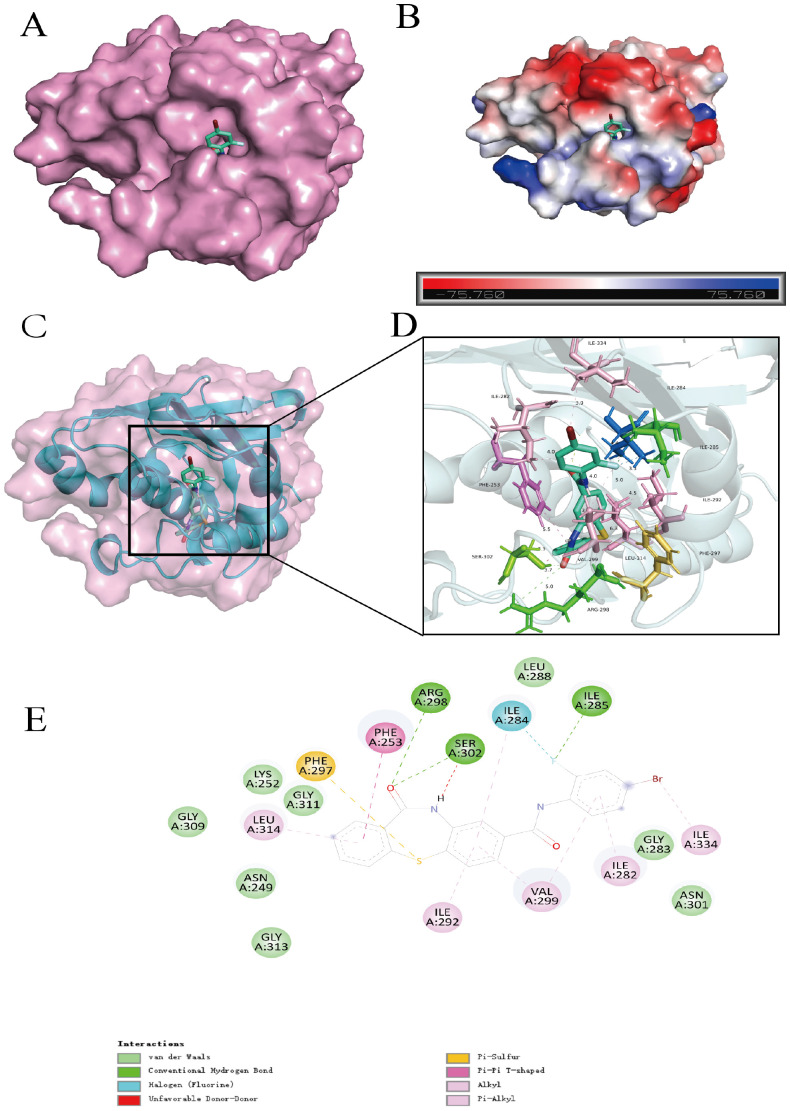
Docking pose and interaction profile of Saeamid-1 in the SaeS kinase domain **(A–E)**. **(A)** Surface representation of SaeS showing the binding location of Saeamid-1; **(B)** electrostatic surface map of the ligand-binding region; **(C)** overlay of surface and secondary structure highlighting ligand placement within the binding cavity; **(D)** close-up view of the binding site illustrating spatial proximity to key surrounding residues; and **(E)** 2D interaction diagram summarizing major contacts, including hydrophobic interactions, hydrogen bonds, and halogen-associated directional contacts.

Docking analysis showed that Saeamid-1 adopted a stable binding pose in the reported ATP-binding pocket of the SaeS kinase domain. The ligand was mainly surrounded by hydrophobic residues, which supported shape complementarity and close packing (**Figures 5A–E**) (Verteramo et al., 2024). The ligand also formed local polar contacts near the boundary of the pocket, and these contacts likely helped maintain its orientation within the binding site (**Figures 5D, E**). In addition, the halogen-substituted group was positioned in a geometry compatible with directional halogen-related contacts (**Figures 5D, E**) (Alisaac, 2025; Viering et al., 2024). Overall, the docked complex displayed a binding pattern characterized by a hydrophobic core together with localized polar constraints. This pose was used as the starting structure for the subsequent molecular dynamics analysis (**Figure 5B**).

### 3.4 Molecular dynamics simulations

**Figure 6:**
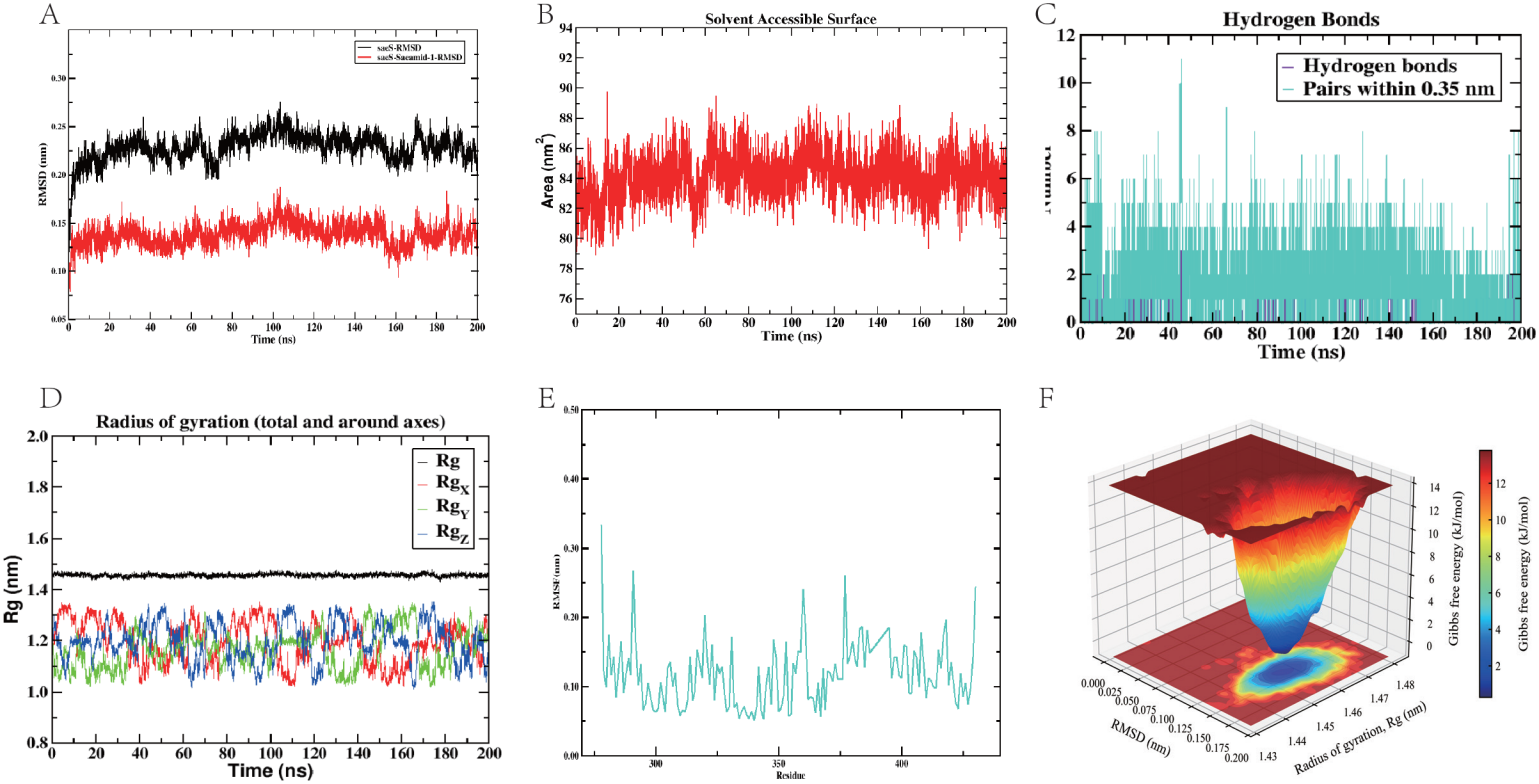
Molecular dynamics characterization of the SaeS–Saeamid-1 complex **(A–F)**. **(A)** Time evolution of RMSD comparing apo SaeS and the SaeS–Saeamid-1 complex; **(B)** time course of solvent-accessible surface area (SASA) associated with the binding region; **(C)** trajectories of protein–ligand hydrogen bonds and the number of atom pairs within 0.35 nm; **(D)** radius of gyration (Rg) and its axial components over time to reflect overall compactness; **(E)** residue-wise RMSF profile to assess local flexibility; and **(F)** free-energy landscape projected onto RMSD and Rg, highlighting dominant conformational basins and clusters.

To assess the stability of the complex formed by SaeS and Saeamid-1 over time, we analyzed molecular dynamics trajectories for both the ligand-bound complex and SaeS in parallel (**Figure 6A–F**). The root-mean-square deviation of the complex increased during the early stage of the simulation and then reached a stable plateau. The plateau values remained lower than those observed for apo SaeS (**Figure 6A**), which is generally consistent with improved conformational stability after ligand binding (Park et al., 2023). The radius of gyration varied within a narrow range and showed no systematic drift during the simulation (**Figure 6D**), while the solvent-accessible surface area associated with the binding region fluctuated within a limited interval (**Figure 6B**) (Park et al., 2023). Taken together, these results supported the stable residence of Saeamid-1 within the SaeS binding region over the simulated timescale.

At the interaction level, persistent hydrogen bonds with high occupancy were not the main stabilizing feature. However, short-range contacts between the protein and ligand were maintained throughout the simulation (**Figure 6C**), which is often consistent with sustained ligand residence within the binding region. Root-mean-square fluctuation analysis further showed that residues near the binding region exhibited relatively restrained fluctuations, whereas distal loop regions remained more flexible (**Figure 6E**) (Park et al., 2023). The free energy landscape also displayed a single dominant basin together with a concentrated conformational cluster (**Figure 6F**). These results supported stable residence of Saeamid-1 and overall conformational stability within the SaeS binding region.

### 3.5 Effects of Saeamid-1 on bacterial growth

**Figure 7:**
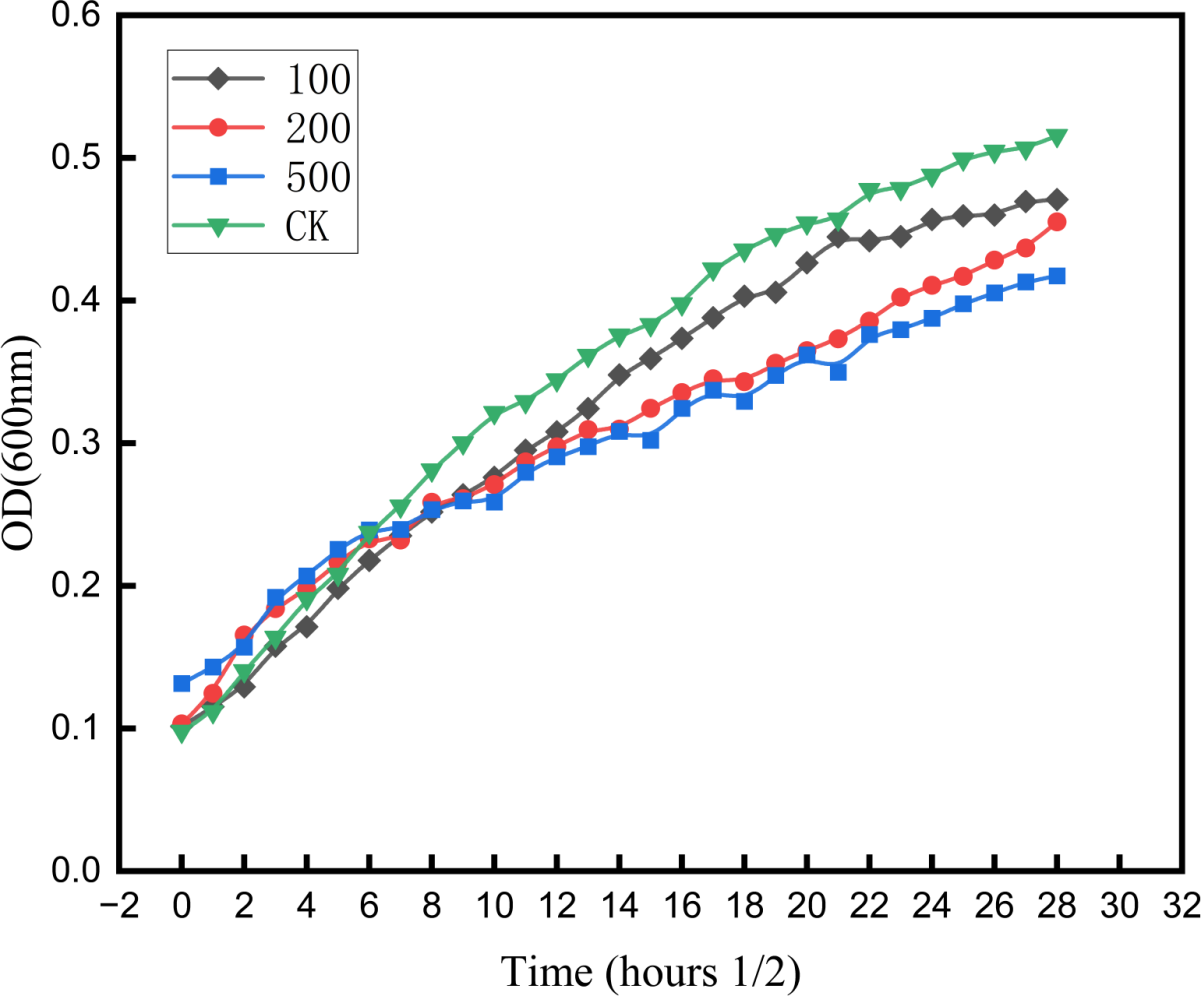
Effect of Saeamid-1 on the USA300 growth curve.

Across the tested concentration range, Saeamid-1 did not cause an abrupt decrease in OD600 or complete growth arrest, and the overall growth curves remained close to those of the control group (**Figure 7**). At higher concentrations, the treated cultures showed a modest delay in growth and a slightly lower final OD600. However, the overall pattern remained consistent with only limited growth inhibition under these conditions. This result supported the interpretation that the downstream virulence-related phenotypes were not primarily caused by overt collapse of bacterial growth (Park et al., 2024).

### 3.6 Hemolysis and biofilm formation

**Figure 8:**
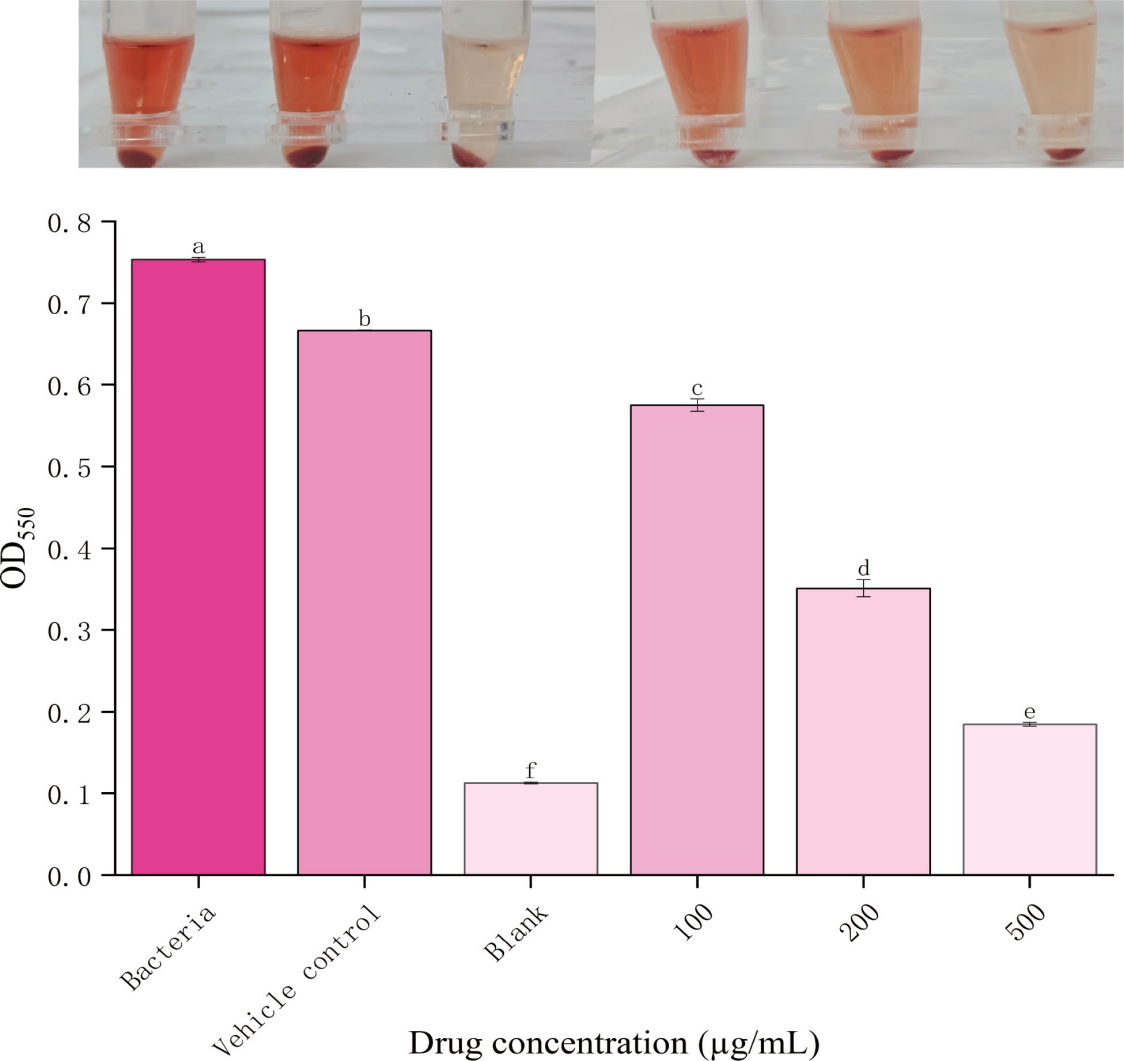
Saeamid-1 decreases hemolytic activity in USA300. Different lowercase letters indicate significant differences among groups at P < 0.05, whereas the same lowercase letter indicates no significant difference.

In the hemolysis assay, the untreated group and the vehicle control showed the highest signals, whereas the blank control remained minimal. Treatment with Saeamid-1 caused a dose-dependent reduction in hemolysis-related readouts (Das et al., 2024). This separation became more evident at higher concentrations, as indicated by the letter or asterisk annotations (**Figure 8**). The representative images showed the same trend. The supernatants became paler, and the erythrocyte pellets remained more intact at higher doses (**Figure 8**). Taken together, these data indicated that Saeamid-1 reduced the hemolytic activity associated with USA300 under the tested conditions (Wang et al., 2021).

**Figure 9:**
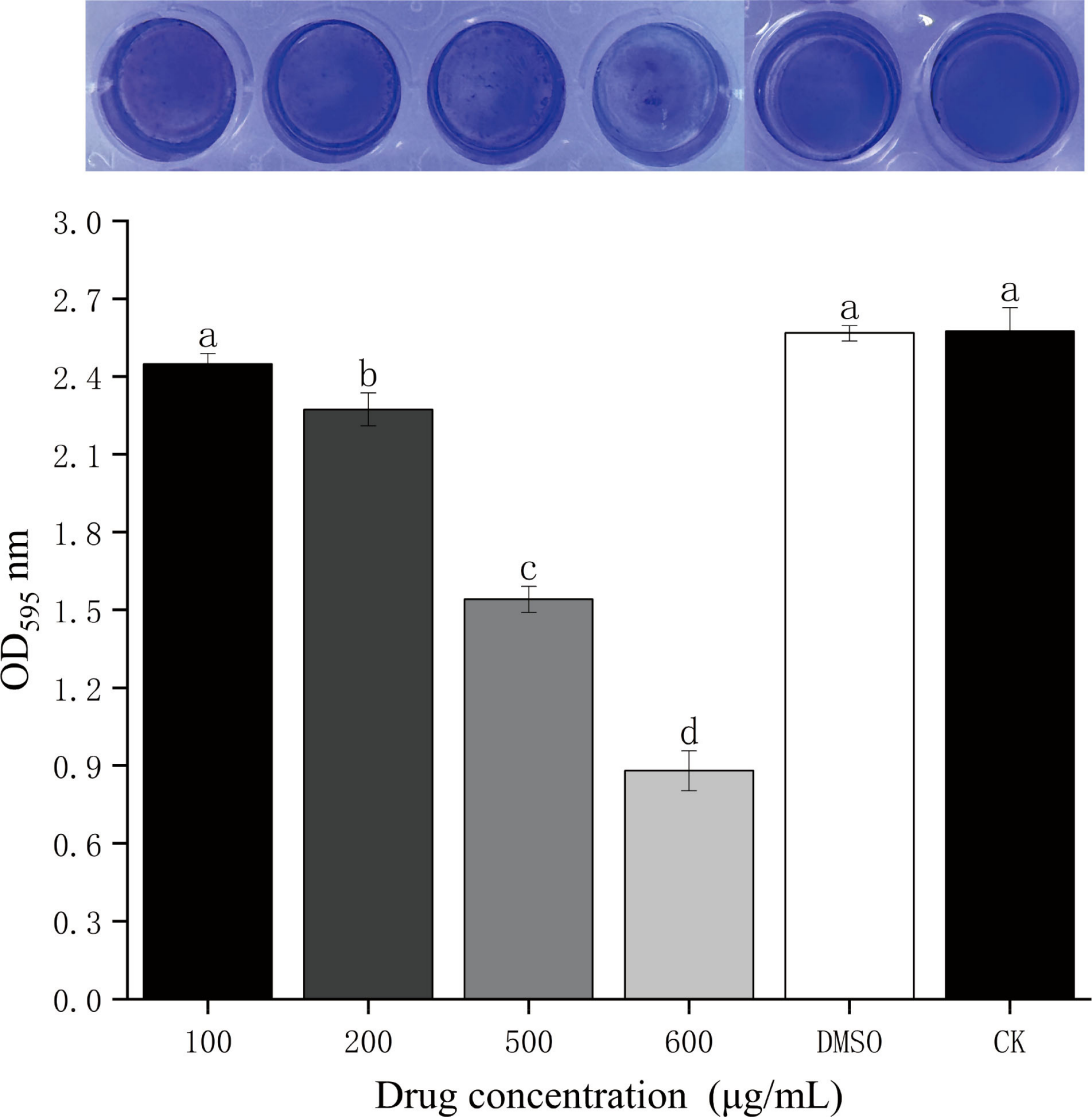
Saeamid-1 reduces crystal violet–quantified biofilm biomass. Different lowercase letters indicate significant differences among groups at P < 0.05, whereas the same lowercase letter indicates no significant difference.

Crystal violet quantification showed that Saeamid-1 reduced USA300 biofilm biomass in a dose-dependent manner. At lower concentrations, the compound produced a downward trend, whereas at higher concentrations it caused a clear separation from the control group, as indicated by the letter or asterisk annotations (**Figure 9**). Together with the hemolysis data, these results indicated that Saeamid-1 reduced both hemolytic activity and biofilm biomass under conditions that did not cause overt collapse of bacterial growth (Zheng et al., 2021) [24].

### 3.7 RT-qPCR

**Figure 10:**
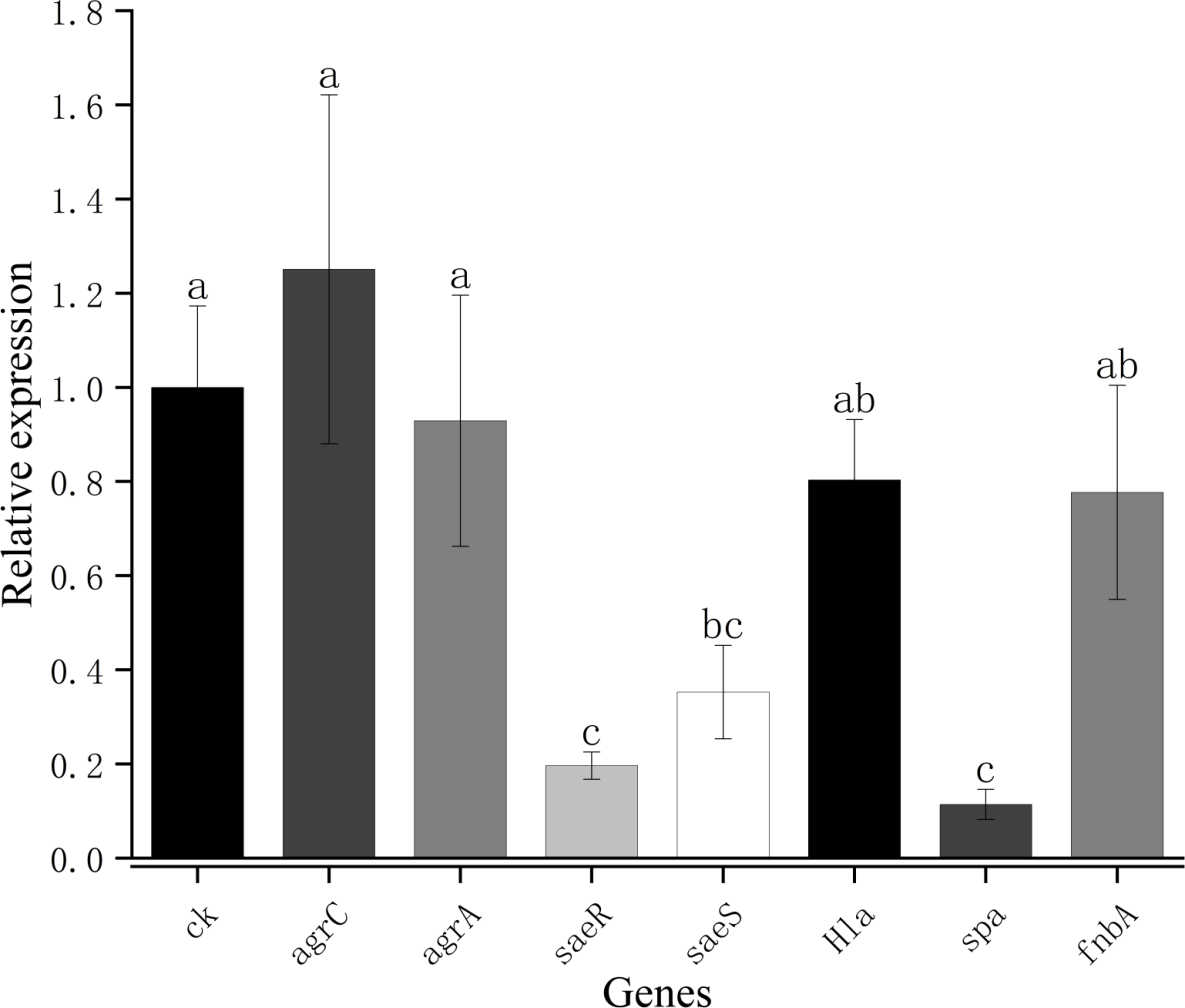
Transcriptional responses of virulence-regulatory genes in USA300 following Saeamid-1 treatment. RT-qPCR relative expression of *agrC*/*agrA*, *saeR*/*saeS*, *hla*, *spa*, and *fnbA.*. Different lowercase letters indicate significant differences among groups at P < 0.05, whereas the same lowercase letter indicates no significant difference.

At the transcriptional level, Saeamid-1 significantly reduced the expression of *saeR* and *saeS*, as indicated by the letter or asterisk annotations (**Figure 10**). This pattern was consistent with reduced activity of the SaeRS virulence regulatory system. In parallel, the surface protein gene spa, which is associated with immune evasion, was also markedly downregulated. By contrast, *agrA* and *agrC* showed only slight increases or remained close to the control group, whereas *hla* and *fnbA* changed within a moderate range (**Figure 10**). Overall, the transcriptional profile was consistent with the phenotypic results, including reduced hemolytic activity and lower biofilm biomass after treatment with Saeamid-1 (Touati et al., 2025).

## 4 Discussion

Saeamid-1 appeared to be a potential antivirulence compound because it reduced hemolytic activity and biofilm biomass while having only limited effects on MRSA growth. This pattern was more consistent with attenuation of virulence than with a conventional bactericidal effect. The structural data supported this interpretation, as molecular docking and molecular dynamics simulations showed that Saeamid-1 remained stably located in the nucleotide-binding region of the SaeS kinase domain, and the protein-ligand complex remained overall stable throughout the simulation. Because histidine kinase activity depends on an ATP-associated catalytic site, this binding pattern was consistent with interference in SaeS signaling (Espinasse et al., 2023; Wang et al., 2024). This interpretation was further supported by recent work showing that phenazopyridine hydrochloride can compete with ATP for SaeS binding, reduce SaeR phosphorylation, and suppress SaeRS-dependent virulence outputs. These findings further support the idea that the ATP-associated catalytic region of SaeS is a druggable antivirulence site. The transcriptional results showed the same trend, because Saeamid-1 significantly reduced the expression of *saeR* and *saeS*, whereas *agrA* and *agrC* changed only slightly. This suggested that Saeamid-1 influenced the SaeRS branch of virulence regulation more strongly than the Agr quorum-sensing system. This distinction was important because SaeRS and Agr are both major regulators in the virulence network, but they do not represent the same signaling pathway. In addition, the decrease in *spa* can be interpreted as a downstream response to SaeRS disruption rather than as direct evidence of SaeRS controlled by itself, because *spa* is regulated by a broader network that included Agr and RNAIII, SarA, SarS, Rot, and MgrA. The phenotypic results also supported this conclusion, since Saeamid-1 reduced hemolytic activity and biofilm biomass under conditions that caused only limited growth inhibition. This finding supports the general concept of antivirulence therapy, in which a compound does not primarily inhibit bacterial growth but instead weakens traits associated with host damage, persistence, and poor treatment outcome. Reduced hemolysis was particularly important because hemolysins and other pore-forming toxins were major contributors to host cell injury. Decreased biofilm biomass was also meaningful, since biofilm growth promoted persistence and was closely associated with poor treatment responses in MRSA infections (Vadakkan et al., 2024; Pant et al., 2022; Mahdally et al., 2021). Recent studies have further extended the significance of these findings by showing that SaeRS contributes not only to toxin production but also to macrophage dysfunction through bacterial clump formation and to host-associated aggregation in joint infection settings through FnbA and FnbB. In this context, the effects of Saeamid-1 on hemolysis and biofilm likely reflected a broader reduction in SaeRS-associated pathogenic behaviors rather than an isolated change in a single phenotype. Overall, these findings suggested that Saeamid-1 mainly acted as an antivirulence agent rather than as a potent antibacterial compound. Targeting SaeS may also complement quorum-sensing-based antivirulence strategies (Vadakkan et al., 2024; Sim et al., 2024), because a compound directed against SaeS could weaken another signaling route linked to exoprotein production, immune evasion, invasive disease, and persistence without relying directly on Agr inhibition (Wang et al., 2024). Overall, these results inferred the SaeRS pathway as a useful antivirulence target and identified Saeamid-1 as a promising lead compound for further investigation. Similar small-molecule antivirulence effects against Staphylococcus aureus biofilm and virulence have also been reported for 3-fluorocatechol (Kim T. et al., 2025).

## 5 Conclusions

This study identified Saeamid-1 as a promising antivirulence candidate against MRSA. Structure-based screening first prioritized this compound from the candidate library, and subsequent molecular docking and molecular dynamics analyses supported stable binding of Saeamid-1 in the ATP-binding region of the SaeS kinase domain. These structural results were consistent with the transcriptional and phenotypic experiments. At the transcriptional level, Saeamid-1 reduced the expression of *saeR*, *saeS*, and the SaeRS-associated virulence gene *spa*, while causing only limited changes in the parallel agr quorum-sensing system. At the phenotypic level, Saeamid-1 decreased hemolytic activity and biofilm biomass without markedly affecting bacterial growth. This pattern indicated Saeamid-1 as an antivirulence agent rather than a strong bactericidal effect. These findings also inferred that the SaeRS pathway represented a useful target for antivirulence development in *Staphylococcus aureus*.

## Data Availability

The original contributions presented in the study are publicly available. The minimal dataset supporting the conclusions of this article has been deposited in Figshare and can be found here: https://doi.org/10.6084/m9.figshare.32898971. Publicly available compound information from PubChem and protein structure information from AlphaFoldDB/UniProt were also used in this study. No genomic sequencing, transcriptomic sequencing, or proteome profiling datasets were generated in this study.
